# A scoping review of computational models on human glucose cerebral metabolism

**DOI:** 10.1177/0271678X261465840

**Published:** 2026-06-24

**Authors:** Parissa Fereydouni-Forouzandeh, Andréanne Michaud, Nicolas Doyon, Simon Duchesne

**Affiliations:** 1 Department of Neuroscience, Laval University, Québec City, QC, Canada; 2 Quebec Heart and Lung Research Institute, Québec City, QC, Canada; 3 School of Nutrition, Laval University, Québec City, QC, Canada; 4 Centre Nutrition, Santé et Société (NUTRISS), Institute of Nutrition and Functional Foods (INAF), Québec City, QC, Canada; 5 Department of Mathematics and Statistics, Laval University, Québec City, QC, Canada; 6 CERVO Brain Research Centre, Quebec Mental Health Institute, Québec City, QC, Canada; 7 Department of Radiology and Nuclear Medicine, Laval University, Québec City, QC, Canada

**Keywords:** Computational, model, metabolism, brain, cerebral, assumptions

## Abstract

Understanding cerebral metabolism is an essential undertaking to address brain health and tackle ambiguities in associated neurodegenerative diseases. Despite the abundance of detailed observational findings on specific functionalities of metabolism, there remains a gap in holistic understanding that could provide a suitable predictive framework, and hence be put to clinical use. Computational models can support such wider goals. Accordingly, we performed a scoping review of computational models on human cerebral glucose metabolism on the PubMed database. We selected 15 models on which we conducted a qualitative assessment of *Verification* based on reproducibility and internal validity; *Validation* for computational and biological relevance, sensitivity analysis and external validity; and *Evaluation*, where we inquired whether the model increased domain knowledge, with the capacity to be extended for improved generalizability. We raised critical shortcomings, including overall poor reproducibility, lack of internal and external validity, and certain unclear metabolic assumptions that compromised biological relevance. We proposed that for a computational model to improve its applicability such as predicting metabolic state through the human lifespan, Verification, Validation and Evaluation (VV&E) issues raised should be addressed, which would facilitate the involvement of disciplines beyond mathematics.

## Introduction

Cerebral metabolism refers to the biochemical pathways by which the brain obtains energy through the breakdown of various metabolites into adenosine triphosphate (ATP), which releases energy following its hydrolysis.^1^ The adult brain consumes 10 times more energy per tissue weight than other organs, making up about a fifth of endogenous energy resources.^2–4^ About 43% of the human’s gray matter energy demand corresponds to synaptic transmission.^5,6^ Other major energetic demands come from microtubular transport of mitochondria towards synapses needed for neurotransmission, maintenance and functionality of active sodium/potassium pump transporters, as well as neurotransmitter biosynthesis.^7^ Among cerebral metabolites, glucose is known as its predominant source of energy,^8^ but there is an unresolved debate regarding the use of lactate during high neural and physical activities, according to the astrocyte–neuron–lactate–shuttle (ANLS) theory.^9–11^ Unlike other organs, the brain does not generally store a significant amount of energy substrates like lipids or proteins,^12^ except occasionally for glycogen.^13^ During prolonged fasting or aging-related metabolic decline, ketones produced by the liver can serve as alternative energy substrates.^14–16^

Metabolic decline in the brain is linked with aging.^17^ It is reported that cerebral metabolic decline is associated with other cellular and molecular impairments such as mitochondrial dysfunction, dysfunctional waste disposal machinery, oxidative damage, telomere attrition, increased neuroimmune response coupled with some inflammatory damage, insulin resistance, neuronal network activity, and more.^18–20^

These changes are exacerbated in neurodegenerative diseases such as Alzheimer’s disease (AD) from early to mid-clinical stages.^21^ There is confusion surrounding whether such metabolic decline begins before AD pathology, other neuropathological events affect metabolic well-being faster than regular aging, or both. Either way, this hypometabolism is believed to increase synaptic loss, neuronal death, and AD-related proteinopathy.^22^ This can aggravate the neuroimmune response and redirect more of the brain’s metabolic focus to itself, leading to a destructive positive feedback loop over the long-term.^14,15^

Understanding these dynamic and complex physiological processes is therefore extremely difficult with mainstream research workflows, yet necessary if we are to generate practical advances in interventions to reduce neurodegeneration. As a result of the rise in advanced wet-lab methodologies, numerous enzymatic or biochemical pathways are being studied rigorously, thus leading to extremely detailed metabolism mechanistic pathways and certain targets for neurodegenerative therapy studies. While discovering metabolic pathways is a huge accomplishment, it is only an observational framework. More importantly, molecular neuro-metabolism is run by thousands of proteins, whose functions we can observe, but not understand, even in their aggregate.^23–25^ Rarely, modulation of a single metabolic protein like insulin shows consistent and effective results all the way up to the organ or systemic level.^26^ However, in the study of neurodegenerative diseases or general metabolic health throughout life, familiarity with hypothesized molecular pathways does not usually allow us to infer simple cause-effect predictions. Computational modeling has recently gained more attention as a more wholesome tool to better understand or try predicting certain outcomes among these multifactorial and multidimensional phenomena, as long as the model is designed vigilantly and reasonably. Computational models of cerebral metabolism can be empirical (i.e. data-driven) or theoretical (i.e. equations-driven). Data-driven models have less human supervision to oversee their theoretical soundness and aim to detect emerging predictive patterns primarily based on datasets.^27^ Typically, the noisier the targeted system, the higher the chance of model overfitting,^28^ which will certainly be the case in the dynamic physiology of the brain. Therefore, data-driven modeling could be a secondary alternative to theoretical modeling, in which a system of mathematical equations describing metabolism is devised by humans.

**Figure 1.**
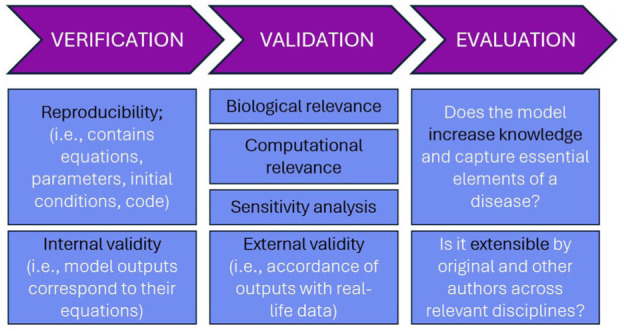
VV&E. Model assessment according to the VV&E paradigm^29^ provides a guide to examine the quality of a model, how representative it is of a natural phenomenon, and whether it contains necessary elements and considerations to be understood and extended by future modelists. This assessment was appropriated to the context of biological computational models. The *Verification* element determines if the model is adequately presented in the article with the correct essential information, allowing it to be reproducible. The *Validation* element checks the overall soundness of the model theoretically and computationally. It assesses if core assumptions and computational methodology make biological and mathematical sense. It also emphasizes the importance of parameter sensitivity through sensitivity analysis, which helps to computationally fine-tune the model with impactful parameters. Lastly, this element addresses if model outputs are externally validated (i.e. with real-life data). The *Evaluation* element assesses if the application of the model can improve our understanding of essential biological and pathological elements, as well as allow one to improve and extend it. In the following sections, we discuss each component of the VV&E elements through a high-level assessment of the models based on our findings. VV&E: Verification, Validation and Evaluation.

In this work, we perform a scoping review of theoretical computational models of human cerebral metabolism, alongside a qualitative assessment of their internal and external validity (Table S1) following the Verification, Validation, and Evaluation (VV&E; for definition of underlined terms, refer to Table S1) quality assurance paradigm (Figure 1).^29^ In this framework, we assessed whether the models were understandable, reproducible, extensible, and translatable across disciplines.

## Methods

### Search strategy

This scoping review was conducted following the 2020 Preferred Reporting Items for Systematic reviews and Meta-Analyses (PRISMA) guidelines^30^ using the Covidence systematic review software (Covidence, Melbourne, VIC, Australia) for abstract screening and full-text data extraction. We searched the National Center for Biotechnology Information PubMed database from inception date up to March 2024 with the following query: “mathematical AND model AND brain AND glucose.”

### Study selection

Articles needed to meet the following general inclusion criteria: (a) written in English, (b) being original (i.e. not a review), (c) including full-text, and (d) not an imaging-only study. Specific inclusion criteria were that the model needed to: (a) target glucose metabolism in the human brain, (b) be completely described, that is, providing all necessary equations, and (c) be reproducible, that is, providing parameters and initial conditions. Following our initial search on PubMed and manual additions, references were downloaded and imported into Covidence, which automatically checked for duplicates. Title and abstract screening were followed by full-text review, according to our inclusion criteria. Title and abstract screenings were conducted by two independent reviewers (SD and PF-F), with full-text data extraction by one reviewer (PF-F).

### Data extraction

We extracted general data including the study’s digital object identifier (DOI) to ensure the full-text was available, whether it was written by a single or multiple authors, author names, countries of publication, study funding sources if disclosed, and the possibility for conflict of interest. Additionally, model-specific data was extracted to expand upon methodological approaches: main model entities; computational approach; whether the sources of parameters and initial conditions (Table S1) were based on either human or animal studies, or both; and whether the model outputs were validated, and if so with literature data or real-life data (Table S1) generated in their own study.

### Bias risk assessment

**Figure 2.**
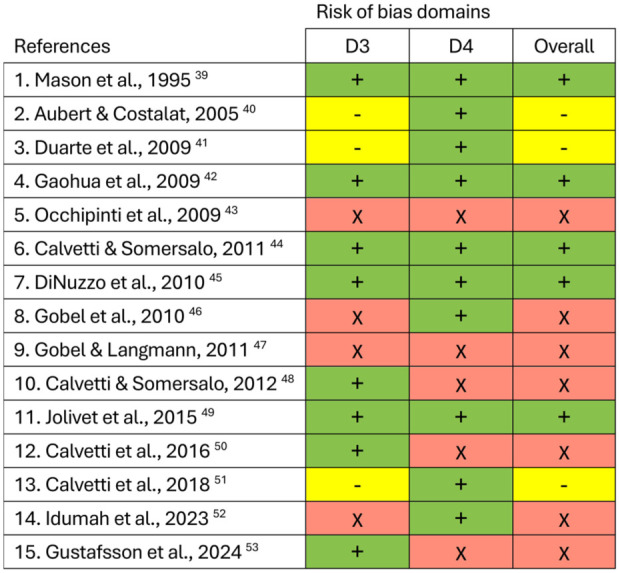
Cochrane risk bias assessment tool, appropriated to the context of metabolic computational models. Using this tool,^31^ we assessed bias risks of each model with respect to D3, corresponding to bias due to missing outcome data. We rated a low concern (+) if authors stated which species their parameters are based on, medium (−) if they were incompletely stated, and high (X) if they were inadequately stated, leading to a high impact bias risk on the model’s relevance and reliability. D4 corresponds to bias due to the measurement of the outcome where a low concern (+) means the model’s basic elements (equations and parameter values, as elaborated in the model completeness section of Figure 3) were adequately provided. A medium (−) and high concern (X) means that certain or a significant portion of these essential elements were missing, respectively, which would impact a model’s verifiability. If at least one domain is medium or high risk, the model is deemed as having an overall medium, or high risk of bias, respectively. D3: domain 3; D4: domain 4.

**Figure 3.**
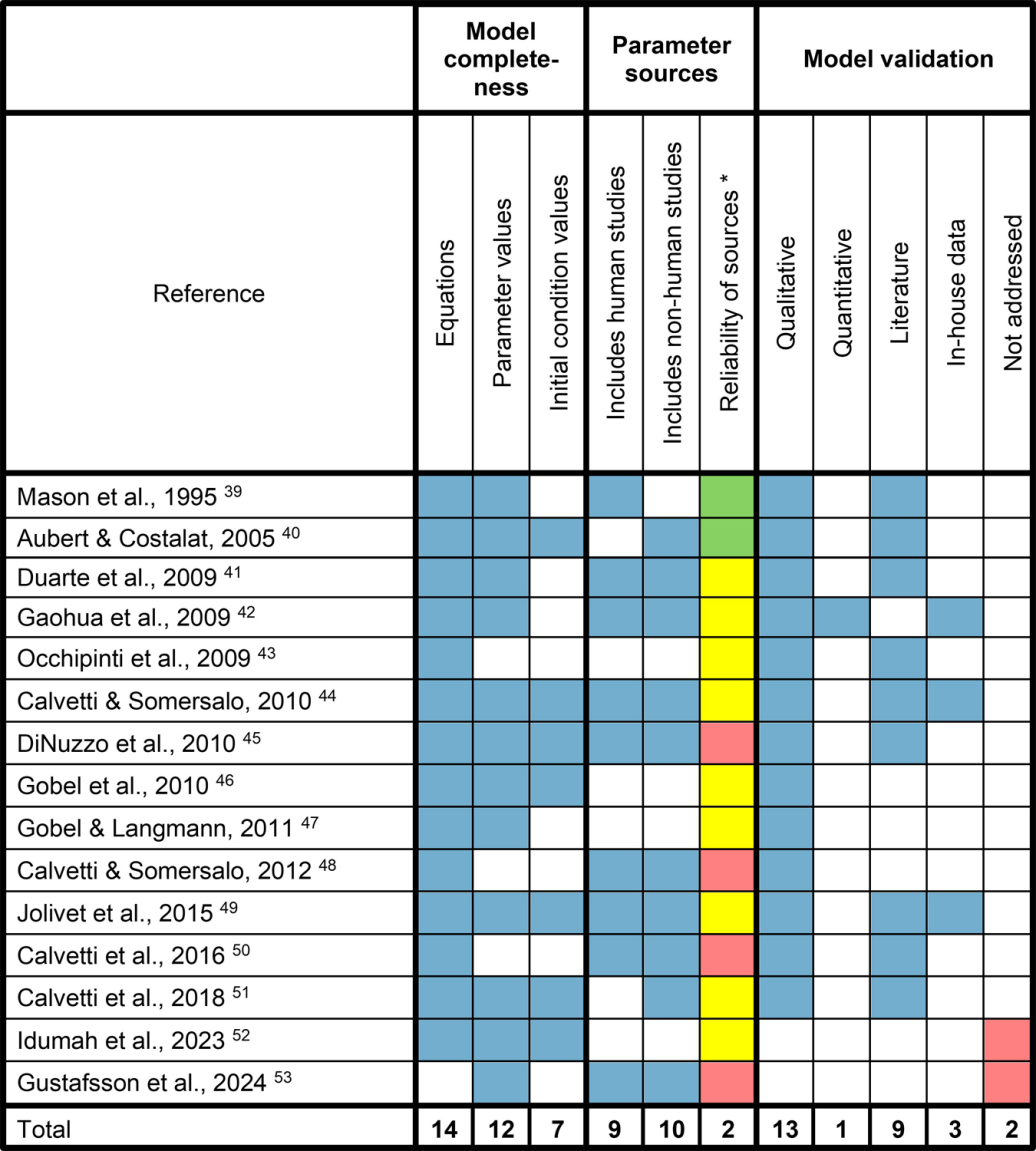
Verification and Validation summary. Model attributes according to the VV&E paradigm are shown as model completeness and parameter sources (both of which belong to the Verification stage of VV&E), and external validation. Blue indicates the presence of an attribute necessary to the VV&E. *Reliability of parameter sources: green = parameter values matched those in their sources, and internally obtained parameters clearly identified (only green cases were counted for “total” row). Yellow = while some references were given, we were not able to verify others because certain internally obtained parameters (i.e. by authors) were not distinguishable from external ones. Otherwise, in the case of external parameters which were modified by the authors, their original values were not provided. Red = If the sources were provided, certain values did not match those in the reference provided, and the authors did not report having modified them.

We used the Cochrane risk bias assessment tool^31^ to perform an assessment. Only two out of five question domains were relevant to computational model articles and are reported here (Figure 2). Domain 3 (bias due to missing outcome data) was rated low if the authors reported on which species (e.g. humans, animals, bacteria) their parameter values were based on, and high otherwise. For domain 4 (bias in measurement of outcome), we assigned a low risk if the model was deemed complete, based on our model completeness estimate (Figure 3), and high otherwise. Knowing that both domains are essential, non-negligible requirements for reproducibility, we assigned an overall high risk of bias if either domain was deemed high-risk.

### Model classification

**Figure 4.**
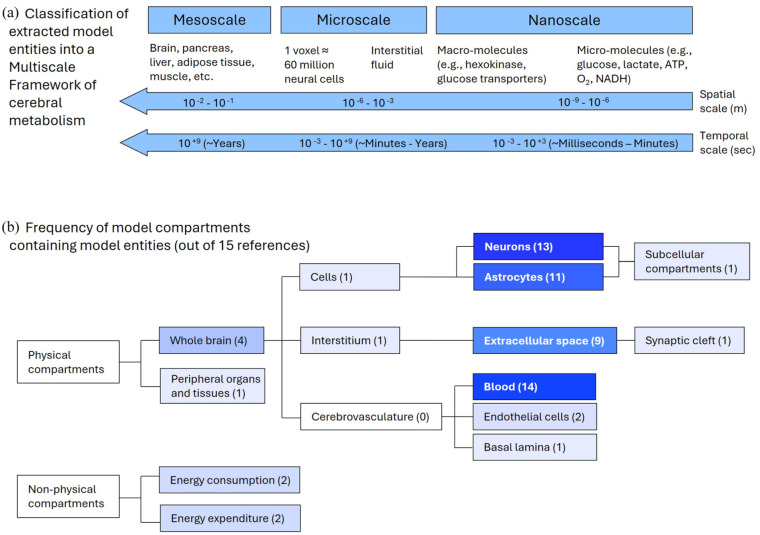
(a) Classification of extracted model entities into a multiscale framework of cerebral metabolism. Examples of entities (see definitions in Table S1) as included in these models are indicated under each scale of our framework. We classified micro and macromolecules as *nanoscale* entities housed in a typical compartment, exhibiting measurable changes in the temporal scale range of milliseconds to minutes. A voxel of neural cells and interstitial space were considered *microscale*, which would show measurable changes within up to years. The “whole brain” and peripheral organs such as the pancreas, liver, as well as adipose, and muscle tissue entities were considered as *mesoscale*, which would be typically tracked with diagnostic medical imaging modalities and (b) Frequency of model compartments containing model entities. Hierarchized compartment names are shown as found in the reviewed articles, followed by the number of articles in which they were accounted for, with darker shades representing higher frequencies. The authors presented their entities of interest by referring to the compartment that houses them. The concept of compartments in these models implied the core assumption of a spatially lumped environment, also known as homogeneous (cf. Table S1), meaning that biochemical reactions occur uniformly throughout that space. The top chosen compartments (i.e. neuron, astrocyte, extracellular space, blood) contained nanoscale entities.

In the current review, we classified metabolic factors within models into different scales, recognizing that cerebral metabolism acts within a multiscale framework (Figure 4(a)).^32^ For example, if a computational model included equations with sub-cellular factors in terms of their size and corresponding temporal scales (e.g. glucose, ATP, hexokinase, glucose receptors, etc., which are housed in a single cell), we grouped them as nanoscale entities (see Table S1). Cellular factors (e.g. neuronal or astrocytic concentration) were termed as microscale, and metabolic factors exhibiting measurable effects on the whole brain or other metabolically related organs (e.g. glucose in the whole brain, fluorodeoxyglucose positron emission tomography or FDG–PET brain scans, or more generic entities like body-wide resources) were considered as mesoscale.

Based on acquired knowledge, and biologically sound assumptions, biochemical and metabolic reactions are usually assumed to take place homogenously in neural environments called compartments (cf. Table S1 and Figure 4(b)), such as neurons and glial cells. Equations link these compartments, relying on fixed parameters and initial conditions.^33^

### Model assessment

In the context of computational models, we examined each model following VV&E assessment elements as described below (Figure 1). The aim was to determine if clear and essential information was provided by the authors to enable cross-disciplinary scientists to understand, verify and expand the model with complementary expertise and perspectives.

#### Verification

This element serves as an internal checkpoint on the availability of basic computational and methodological details for model relevance and reproducibility. We assessed whether basic computational details were provided, which are essential to allow reproducibility or develop the model further. This included making the source code available; providing the complete list of equations, parameters and initial conditions; providing the source of parameter values, specifying whether human or non-human (e.g. animals, bacteria, yeasts) findings were used to estimate parameters. A model should also be internally valid, meaning that reproducing the model should output the same results as the simulation plots provided by the authors.^34^ Models are internally validated on a similar dataset as that used for training. Since internal validity would usually be conducted externally (i.e. not necessarily by original authors, in the same article), it is overviewed in the Discussion section.

#### Validation

This element looks at the overall model design in whether the authors provided clear explanations of their core assumptions that form the pillars of their model and impact the validity of its outcomes. We emphasized the importance for assumptions to be stated and justified as it can have a strongly pivotal role in the quality or reliability of the outputs. Our first focus was on biological relevance, based on the soundness of core theoretical assumptions and how realistically they would represent the true nature of cerebral metabolism in the chosen scale(s). This assessment was helped when models included a conceptual diagram, which illustrated the central thesis or main contribution and the scope of metabolic pathways for a given model. Conceptual diagrams also identified the compartments of the model. We assessed the effectiveness of these diagrams in helping the reader understand the model’s core assumptions, the layout of the equations, and which spatial and temporal scales were in focus. To assess the complexity of the models, we assumed that the diagrams reflect how minutely the metabolic pathways of interest were formulized, and that more complex diagrams correspond to higher computational load. We also assessed methodological and computational relevance, with a focus on whether their application was adequate in the neuro-metabolic environment. Given the complex and poorly understood nature of cerebral metabolism, we looked for the presence of sensitivity analysis, which is crucial for identifying more influential parameters whose modulations lead to strongest output fluctuations.^35^ We then looked for the presence of external validation of model outcomes with real-life metabolic data that represent the model’s variables as closely as possible,^36^ as well as how adequately this was reported and interpreted. External validation involves assessing model results on an independent dataset, as a way to assess the quality of the model’s prediction.

#### Evaluation

It assesses if the model can increase our overall knowledge of the metabolic mechanism and help capture new elements of a related disease, with a particular focus on their applicability in AD diagnostics. It also aims to determine if sufficient, clear, and adequate information, particularly stemming from the Verification and Validation prerequisite elements, will enable other researchers to extend the model and improve its predictability. We thus highlight that a successful model, with regards to the VV&E assessment, is accessible across disciplines and applicable for relevant fields such as biomedicine. The evaluation step is performed in the Discussion section of this article.

## Results

### Search results

**Figure 5.**
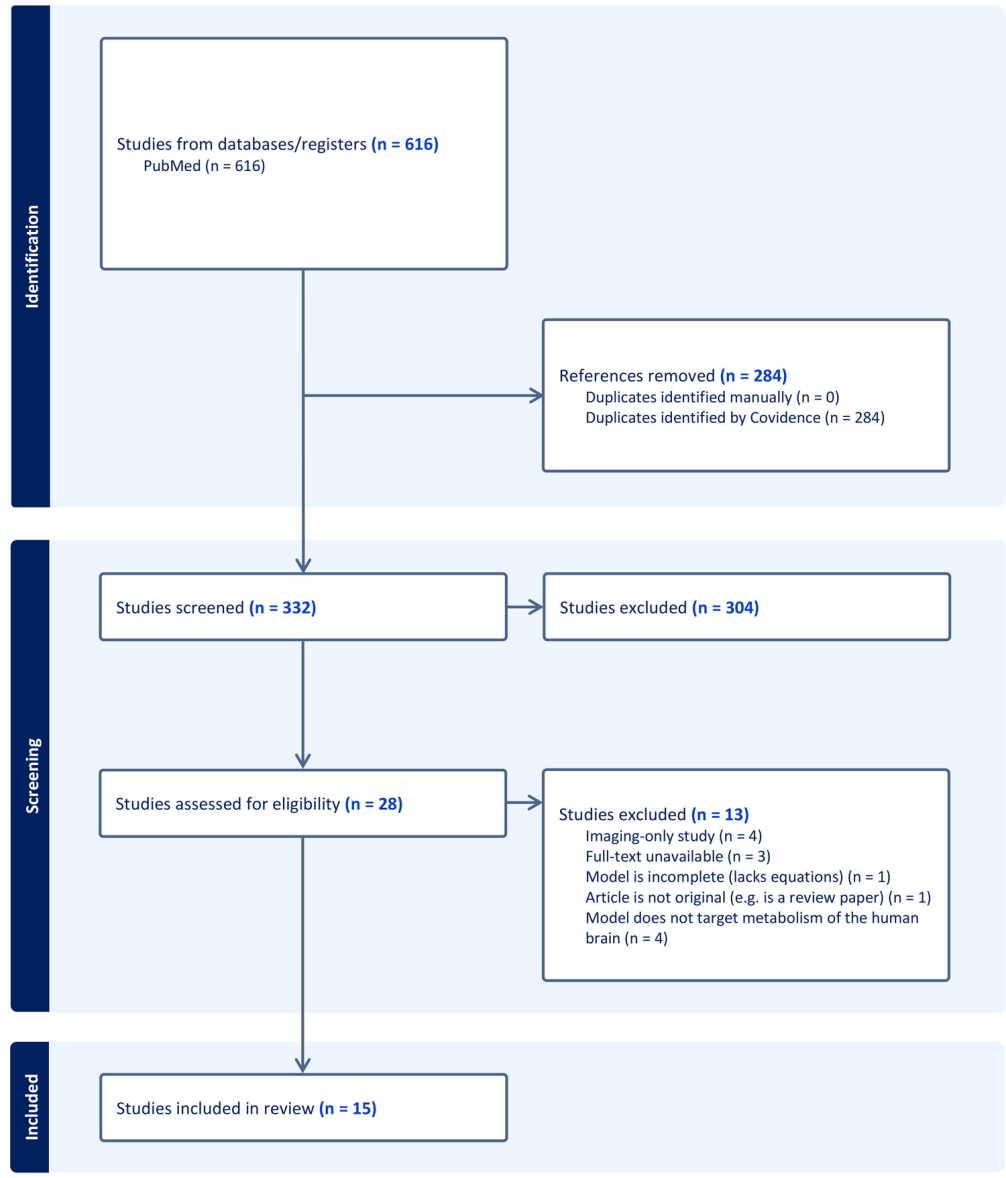
PRISMA diagram. This summarizes the study selection process using Covidence software. Search words were “mathematical,” “model,” “brain,” and “glucose.” Fifteen articles were extracted for this review from 332 initial search results.

Following our initial search on PubMed, 332 references were downloaded and imported into Covidence (Figure 5). Title and abstract screening based on inclusion criteria yielded 28 articles that moved on to the full-text review, after which 15 articles underwent data extraction, summarized in Figures 2 and 3.

### Publication origins

Ten different groups authored the 15 publications. According to the research institution of first authors, three groups from the United States published the majority of the articles (Tables 1 and 2). Two papers originated from Germany. Six other first authors each originated from France, Italy, Japan, Sweden, Switzerland and the UK.

**Table 1.** Key characteristics of each model.

Article reference	Model scales	Compartments containing model entities	Type of model diagram	Model scope based on diagram	Main pathways of interest
Mason et al.^37^ (Simultaneous determination of the rates of the TCA cycle, glucose utilization, α-ketoglutarate/glutamate exchange, and glutamine synthesis in human brain by NMR.)	• Nano	• Neuron • Blood	• Biochemical	• High-level canonical pathways • Glutamate turnover as proxy for brain glucose metabolism	• Glycolysis • Citric acid cycle • V-cycle
Aubert and Costalat^38^ (Interaction between astrocytes and neurons studied using a mathematical model of compartmentalized energy metabolism.)	• Nano	• Neuron • Astrocyte • Extracellular space • Blood (cerebral vein and capillary)	• Biochemical	• Compartment-wise • High-level canonical pathways • Emphasis on NADH exploring role in the astrocyte-neuron lactate shuttle	• Aerobic glycolysis • Anaerobic glycolysis • Astrocyte-neuron lactate shuttle • Creatine-phosphate system • Oxidative phosphorylation
Duarte^39^ (Steady-state brain glucose transport kinetics re-evaluated with a four-state conformational model.)	• Nano	• Neuron • Astrocyte • Blood • Endothelium	• Conceptual	• Glucose transporter conformation dynamics	• Aerobic glycolysis • Anaerobic glycolysis • V-cycle • GLUT1 alternating conformation kinetics
Gaohua and Kimura^40^ (A mathematical model of brain glucose homeostasis.)	• Nano • Meso	• Neuron • Extracellular space • Blood	• Regulatory	• Compartment-wise • Assumes brain as controlled object, rest of body as actuator • Regulation by the brain to maintain body-wide glucose homeostasis	• Kinetics of glucose–insulin–glucagon regulatory system
Occhipinti et al.^41^ (Astrocytes as the glucose shunt for glutamatergic neurons at high activity: an in silico study.)	• Nano	• Glutamatergic neuron • Astrocyte • Lumped blood and extracellular space	• Biochemical	• Compartment-wise • Detailed canonical pathways	• Aerobic glycolysis • Anaerobic glycolysis • Astrocyte–neuron lactate shuttle • Malate–aspartate shuttle • Creatine–phosphate system • Glyoxylate cycle • V-cycle • Oxidative phosphorylation
Calvetti and Somersalo^42^ (Dynamic activation model for a glutamatergic neurovascular unit.)	• Nano • Micro	• Neuron • Astrocyte • Extracellular space • Synaptic cleft • Blood (capillaries)	• Biochemical	• Compartment-wise • High-level canonical pathways	• Aerobic glycolysis • Anaerobic glycolysis • Astrocyte–neuron lactate shuttle • V-cycle • Creatine-phosphate system • Oxidative phosphorylation • ATP hydrolysis
DiNuzzo et al.^43^ (Changes in glucose uptake rather than lactate shuttle take center stage in subserving neuroenergetics: evidence from mathematical modeling.)	• Nano	• Neuron • Astrocyte • Blood–brain barrier	• N/A (no diagram)	• N/A	• Aerobic glycolysis • Anaerobic glycolysis • Astrocyte–neuron lactate shuttle • Sodium–potassium ATPase pump regulation of neural sodium influx
Göbel et al.^44^ (Compact energy metabolism model: brain controlled energy supply.)	• Nano	• Energy resource in peripheral organs • Blood glucose • Blood insulin (control signal) • Brain ATP • Ingested energy • Appetite (control signal)	• Regulatory	• Compartment-wise • High-level energy fluxes • Regulatory relationships	• Glucose fluxes • Insulin and appetite signaling • Ingestion momentum • Energy competition between brain and peripheral organs
Göbel B and Langemann^45^ (Systemic investigation of a brain-centered model of the human energy metabolism.)	• Nano	• Energy resource in peripheral organs • Blood glucose • Blood insulin (control signal) • Brain ATP • Ingested energy • Appetite (control signal)	• Regulatory	• Compartment-wise • High-level energy fluxes • Regulatory relationships	• Glucose fluxes • Insulin, appetite, and ingestion signaling • Ingestion momentum • Energy competition between brain and peripheral organs
Calvetti and Somersalo^46^ (Ménage à trois: the role of neurotransmitters in the energy metabolism of astrocytes, glutamatergic, and GABAergic neurons.)	• Nano	• Glutamatergic neuronal mitochondria • GABA-ergic neuronal mitochondria • Astrocytic mitochondria • Cytosol • Extracellular space • Blood	• Biochemical	• Compartment-wise • Canonical pathways • Reaction fluxes and transport rates within and between compartments	• Aerobic glycolysis • Glyoxylate cycle • Malate–aspartate shuttle • Alanine–pyruvate shuttle • Oxidative phosphorylation • Citric acid cycle
Jolivet et al.^47^ (Multi-timescale modeling of activity-dependent metabolic coupling in the neuron–glia–vasculature ensemble.)	• Nano	• Neuron • Astrocyte • Extracellular space • Blood (cerebral vein and capillary)	• Biochemical	• Compartment-wise • High-level canonical pathways • Emphasis on NADH used as proxy for metabolism	• Aerobic glycolysis • Anaerobic glycolysis • Astrocyte–neuron lactate shuttle • Oxidative phosphorylation • Creatine-phosphate system
Calvetti et al.^48^ (Uncertainty quantification in flux balance analysis of spatially lumped and distributed models of neuron–astrocyte metabolism.)	• Nano	• Neuron • Astrocyte • Extracellular space • Blood • Whole brain	• Biochemical	• Compartment-wise • High-level canonical pathways • Transport rates and fluxes within and between compartments	• Aerobic glycolysis • Anaerobic glycolysis • Astrocyte–neuron lactate shuttle • V-cycle
Calvetti et al.^49^ (A computational model integrating brain electrophysiology and metabolism highlights the key role of extracellular potassium and oxygen.)	• Nano	• Neuron • Astrocyte • Extracellular space • Blood	• Conceptual	• Compartment-wise • Very high-level energy fluxes	• Aerobic glycolysis • Anaerobic glycolysis • Astrocyte–neuron lactate shuttle • Citric acid cycle • Oxidative phosphorylation • Creatine-phosphate system • ATP hydrolysis
Idumah et al.^50^ (A spatially distributed model of brain metabolism highlights the role of diffusion in brain energy metabolism.)	• Nano	• Neuron • Astrocyte • Extracellular space • Blood	• Conceptual	• Compartment-wise • Multidomain diffusion model of metabolites between compartments	• Aerobic glycolysis • Anaerobic glycolysis • Citric acid cycle • Oxidative phosphorylation • V-cycle • ATP hydrolysis
Gustafsson et al.^51^ (Brain energy metabolism is optimized to minimize the cost of enzyme synthesis and transport.)	• Nano	• Neuron • Astrocyte • Extracellular space • Blood	• Conceptual	• High-level canonical pathways • Sub-cellular energy costs	• Aerobic glycolysis • Anaerobic glycolysis • Astrocyte–neuron lactate shuttle • Oxidative phosphorylation

**Table 2.** Country distribution of first authors.

Country	*N* papers
US	7
Germany	2
France	1
Italy	1
Japan	1
Sweden	1
Switzerland	1
UK	1
Total	15

### Bias risk assessment

Figure 2 shows domain risk and overall bias risk for all articles. Nearly half of all articles were deemed highly biased. There was less bias risk in domain 4 pertaining to basics of model completeness, but considerably higher in domain 3 which was the transparency on reporting parameter sources.

### Verification

#### Reproducibility

The basic reproducibility elements were determined based on the availability of certain details as in Figure 3. The model source code was not provided except for one article.^51^ Most authors provided all model equations, except for one.^51^ Twelve authors provided parameter values,^37–40,42–45,47,49–51^ and, contrary to our expectation, initial conditions were not explicitly stated in any of the reports. However, considering that most models assumed steady-state conditions, we presumed that basal or resting state values count as initial conditions. Even so, such values were quite scattered in equations or in the text and difficult to identify.

One model used parameters taken from human-only data.^37^ Eight models’ parameters came from a mix of human and non-human experimental findings^39,40,42,43,46–48^ and two models only used parameters from non-human findings.^38,49^ Notably, parameter sources were scarcely or not reported in four cases as marked in red in Figure 3,^43,46,48,51^ and a majority (i.e. nine of 15) had parameters whose sources are unverifiable as explained in the of description Figure 3 for the yellow category.^39–42,44,45,47,49,50^ Meanwhile, only two models provided verifiable and reliable sources for their parameters.^37,38^

### Validation—Biological relevance

#### Diagrams

Many authors provided cellular and molecular mechanism diagrams.^37,38,41,42,46–48^ These generally showed biochemical pathways between metabolic entities. Overall, models with a biochemical diagram relied on flux balance analysis (Table S1), which is primarily based on stoichiometric or molar ratios according to each reaction’s mass balance equation. This type of diagram pointed to the molecular pathways of interest but did not show all of the entities used in the model. Some,^40,44,45^ but not all^41,42,48,50^ models which focused on regulatory pathways provided relationship diagrams between entities diagrams, which contributed to the biological realism of the model and allowed one to track these relationships in its equations. Alternatively, some models depicted a conceptual diagram,^39,49,51^ or simply an overview of the compartments,^40,50^ summarizing the theoretical focuses of the model without including all of its entities. Finally, some illustrated their model only partially, mainly to depict rate equations of metabolites.^39,40,43,47^

#### Compartments

Compartments in all models rested on the assumption that their environments were homogeneous, meaning that metabolic reactions occurred uniformly throughout all individual constituents (e.g. all neurons behave similarly). Most included four to five compartments, with the highest being 19.^40^ The main compartments in common were neurons, astrocytes, the extracellular space and blood.^38,42,46–51^ The Mason model^37^ only included neuron and blood compartments. The blood compartment was the most commonly chosen, as shown in Figure 4. Three models considered the blood-brain barrier (BBB) components as compartments.^39,41,43^ The Göbel models^44,45^ stand out as they included non-physical compartments to account for ingestion and energy resources. However, variables representing the latter compartments were unitless.

#### Scales

Regarding spatial scales, all models included entities at the nanoscale (Table S1); one included entities at the microscale^48^; while one model also included what could be construed as mesoscale entities.^40^ There were hints of microscale computations in three models since they mentioned their adaptability to BOLD fMRI signals,^38,42,47^ but only one of them^42^ clearly stated relevant details (i.e. the model computes metabolism in one cortical gram which corresponds to one voxel, or at least 250,000 neural cells^52,53^). The Gaohua model^40^ includes mesoscale compartments given their consideration of whole organs, namely the brain and peripheral organs. Some compartments of the Göbel models^44,45^ (daily energy consumption and expenditure) could not be physically considered as compartments, which we called non-physical (Figure 4(b)).

Based on the information provided in the papers, we have found two truly hybrid models, meaning they represent more than one scale according to our abstraction levels: the 2011 Calvetti model^42^ covers the nanoscale and microscale, and the Gaohua model^40^ covers the nanoscale and mesoscale (with considerations of metabolism in the brain and peripheral organs).

#### Complexity

The main chosen metabolic pathways touched upon glycolysis, oxidative phosphorylation, the tricarboxylic acid cycle, the pentose phosphate pathway, and glutamate recycling as in the V-cycle. The theoretical scope of the models was quite variable. Some models focused on cerebral metabolism in general, while others emphasized a sub-process such the regulatory kinetics of glucose carriers,^39^ or a model able to couple electrophysiology and cerebral metabolism.^49^ This influenced their choice of compartments. Given that all 15 models included the nanoscale, an entity was usually considered as a micromolecule (e.g. glucose, O_2_, ATP) or macromolecule (e.g. metabolic enzymes).

For highly complex models, which was a popular tendency, this choice was not justified in terms of the trade-off of introducing a higher computational burden into the system. Meanwhile, computational complexity was associated with higher model uncertainty, which was quantified with a stochastic method like Bayesian flux-balance analysis.^41,48^ The most complex model^41^ contained about 30 computed entities in each neural compartment (i.e. total of about 60 in neuron and astrocyte combined). On the other hand, the Göbel models^44,45^ were the least complex ones, with only five entities.

### Validation—Methodological relevance

#### Computational approach

**Figure 6.**
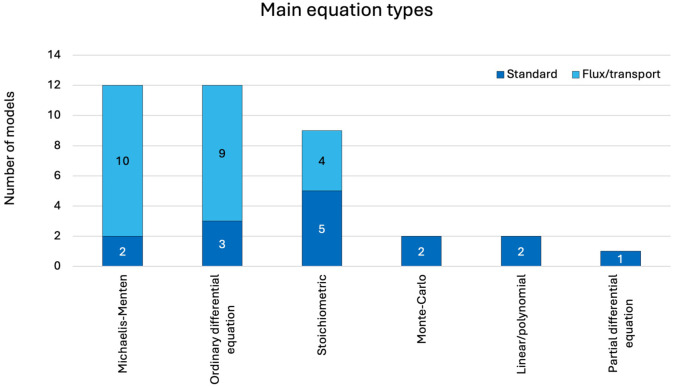
Types of equations within the models. To differentiate between equations obtained from flux-balance analysis methods, we grouped the type of equations for each model as standard or flux-based. Two models contained standard Michaelis–Menten equations.^41,47^ Eleven models contained flux or transport-based Michaelis–Menten equations.^37–40,42,44,45,47,49–51^ Three contained standard ordinary differential equations.^44,45,50^ Ten contained flux or transport-based ordinary differential equations.^37–44,47,49^ Five contained standard stoichiometric equations.^41,42,46,48,50^ Five contained flux or transport-based stoichiometric equations.^37,42,46,48,49^ Two contained Markov–Chain Monte–Carlo equations.^41,50^ Two contained linear or polynomial equations,^44,45^ and one contained partial differential equations.^50^

As shown in Figure 6, the content of the articles predominantly discussed mathematical choices and assumptions. Thirteen models included Michaelis–Menten equations.^37–42,44,45,47,49–51^ Thirteen models devised it with ordinary differential equations.^37–42,44,45,47,49–51^ Ten models included stoichiometric equations.^37,41,42,46,48–50^ Two models included prior probabilities, namely, Markov–Chain Monte–Carlo equations.^41,50^ One model contained partial differential equations.^50^ Lastly, two models included other types, such as linear or polynomial equations.^44,45^

#### Sensitivity analysis

Only two papers,^37,45^ performed sensitivity analysis by parameter perturbations. A cursory search in the PubMed database suggested that other groups have not externally conducted a sensitivity analysis on any of the models.

### Validation—External validation

As shown in Figure 3, 13 models performed external output validation,^37–43,45–49^ but nearly all of them validated only qualitatively (except the Gaohua model^40^). Out of these, five models did not report what type of data they were validating with and only reported that their findings are in line with those in the literature.^44–46,50,51^ Nine models mentioned literature sources,^37–39,41–43,47–49^ and three used real-life data obtained internally.^40,42,47^ Lastly, we noted that two models did not mention anything about validation.^50,51^

## Discussion

### Summary of findings

Following the VV&E tool^29^ (Figure 1), we did a qualitative assessment of the 15 published models of cerebral metabolism. Overall, we found that the models were more deeply focused on discussing mathematical choices and techniques. Meanwhile, there was much less rigor in explaining the thought process that led to the model’s design on a biological level. However, a reproducible model concerning multiple fields of expertise should be understandable by all corresponding disciplines. Considering that computational neurobiology is a multidisciplinary collaboration, it becomes essential for modelists to consistently rationalize methodologies for all future contributors so that other mathematical experts can verify computational choices, while conversely, neurobiologists can verify biological choices and assumptions. This level of justifiability was generally missing in the reviewed articles, especially on the metabolic and physiological sides. In the following sections, we elaborate on elements of the VV&E to overview strengths and weaknesses of the models in terms of reproducibility, relevance and potential for becoming appliable to the biomedical field.

### Biases—Controllable

Models seemed to follow a certain status quo template, namely in the overall model presentation, biological assumptions, previously debated topics (e.g. ANLS theory), and computational approaches. Adequacy in biological assumptions was an area which lacked the most rationalization.

#### The bandwagon bias towards nanoscale models

Nanoscale models were the most popular choice not only in this review, but also in an earlier one on Alzheimer’s disease models.^54^ With advances in molecular medicine research, traditional and symptom-based studies of human physiology are quickly shifting towards molecular methodologies.^55^ As mathematical experts, in most cases, computational model designers focusing on human neuro-metabolism tend to refer to such recent findings, which we believe causes a “bandwagon” bias, and, as we also observed in this work, leads to a disproportionately high number of nanoscale models.

Further, we noted that most models were intended for a mathematical audience, while readers with neurobiological expertise would need more contextual rationales for how each equation, or groups of equations are devised, and to know of any related assumption. As a result, some essential biological information was missing.

Furthermore, a significant portion of the metabolic models tested the highly debated ANLS theory, but results were almost equally split between supporting and not supporting the theory. Importantly, we noted that there were no critically innovative ideas mentioned for future works to try and address or better understand these conflicting results.

### Biases—Uncontrollable

In the context of the chosen computational approaches, some sources of uncertainty are uncontrollable and could make reproducibility much harder. Models largely relied on metabolic flux reactions according to stoichiometric mass-balance equations and boundary conditions that would define the constraints of the steady-state condition. Although not always mentioned clearly, it seemed to indicate that the popular flux-balance analysis method is based on Michaelis–Menten kinetics. However, we cannot forget that these related parameters are taken from experimental findings in tightly controlled laboratory environments.^56^ There is a lack of standardized translational techniques to situate non-human findings into a human’s physiology, which is an important uncontrollable bias that most authors encountered when trying to obtain biologically reliable parameters.

### Verification—Reproducibility

As summarized in Figure 3, equations and parameters were included in most models, but we observed that initial conditions were difficult to identify because most models set their equations in a steady-state framework and therefore referred to initial conditions as steady-state or basal values. As one of the likely consequences of the bandwagon effect, models did not consistently report the source or methods of obtaining their parameters, which compromises the trustworthiness and reproducibility of the model as a whole.

### Verification—Internal validity

For a model to enter the development cycle, it is important to verify if it contains the equations that match the plotted outputs, so that we know we are dealing with the right equations and parameters, which may not always be the case. Given that models do not necessarily need to be internally verified by the original authors specifically, this stage of the VV&E does not apply to the current models.

### Validation—Biological relevance

To assess model relevance, the clarity of assumptions has a crucial role. Assumptions can significantly reflect the design path and interpretability of the model, with pivotal impacts on its predictability. In general, assumptions were scattered throughout the text, usually without much justification.

For example, the popular steady-state and associated flux-balance analysis approaches consist of a set of stoichiometric equations and enzymatic rates that represent the chosen metabolic pathways. Certain rates were taken from the literature with a derivation of the Michaelis–Menten method, and others were internally generated by different estimation methods. We noted inconsistencies in the way parameters were explained, as it was not always clear which were taken from the literature as is, which were then modified, and which ones were inferred internally by the authors (Figure 3).

Most models represented a complex version of metabolic pathways, which in itself led to a lengthy list of nanoscale equations, introducing higher computational burden and uncertainty. Meanwhile, it was never justified why modeling at this level of complexity is more promising than optimizing the number of equations while including other scales, which may have made them more physiologically comprehensive.

Besides the importance of justifying the biological relevance of models, a key question is: how are these brain metabolism models ultimately different from one another? We will reiterate two model characteristics to address this.

Model diagrams, as well as the load of equations and parameters help characterize their complexity and granularity. Additionally, the selection of specific entities and metabolic sub-pathways indicate the mechanistic scope of each model.

Regarding the scope, the introduction sections were generally a good place to find this information, whether it was on a highly debated topic of brain metabolism such as the ANLS hypothesis, or a specific, yet impactful process of the mechanism such as the glucose gate or carrier system on the membranes of neural cells. On another note, most models chose a highly granular version of the metabolic mechanism and subsequently discussed statistical approaches to tackle the ensuing uncertainty. We speculate that for those cases, the authors assumed that a high level of granularity would enhance the model’s predictive power more than the uncertainty would compromise it. Certain models^40,44,45^ had different levels of granularity, meanwhile, they included information from other organs to consider more comprehensively metabolic relationships between the brain and the rest of the body.

### Validation—Methodological relevance

We observed rigorous mathematical analyses and efforts to mitigate the burden of high dimensional computations. Since most models included a high number of entities at the nanoscale, authors found it challenging to handle the resulting uncertainty. As an attempt to re-simplify the system and its assumptions, metabolic pathways were grouped into homogenized compartments; most commonly a single *neuron* and *astrocyte* surrounded by *blood* and *extracellular space* (Figure 4(b)). In models with complex (i.e. entity-dense) versions of the metabolic mechanism, one way to optimize the number of equations and computational burden could be to add metabolic rates in each pathway branch.

Because it is difficult to access reliable and sufficient data for the numerous parameters of the models,^57^ the authors tried to reduce their dependence on external parameters. This was the main reason why many of them used steady-state methodology. This can provide an optimized range of unknown parameter values and reaction rates based on a series of constraints and assumptions in the system.^58,59^ From a mathematical point of view, this approach protects the model from unreliable parameters in a manageable vectorized system. However, this heavily depends on which stoichiometric equations are chosen and the way in which entities are compartmentalized, as one paper pointed out.^42^ Despite these computational challenges noted by the authors, they did not justify why they initially decided to choose a complex version of cerebral metabolism, instead of designing a simpler, but still representative version of this system.

Michaelis–Menten kinetics were stated and applied in five models.^40,42,43,49,50^ Several models included sodium-potassium pumps in the application of Michaelis–Menten. However, this method is not compatible with these transporters and instead meant for simpler substrate-enzyme interactions.^60^ Alternatively, the authors could have used the Albers-Post model for estimating parametrized rates of such transporters.^60^

Overall, whether model complexity was high or low, justification of the methodological approaches in the context of neurobiology and an adequate comparison with other relevant approaches were quite limited.

#### Sensitivity analysis

Mathematical models should undergo several rounds of improvement as it is unrealistic to expect they will accurately reflect the natural system on the first shot. Besides external validation, it is crucial to examine how changes in parameter values impact the outputs of the model. Therefore, this method, known as sensitivity analysis, is a non-negligible, determining stage in the model development cycle. Despite its importance in this cycle, only two of 15 models performed this analysis. Given that many of the authors expressed difficulty in finding biologically reliable parameters, sensitivity analysis would help them identify the ones with the highest impacts on the outputs of the model. The authors can then narrow their focus and improve the reliability of those parameters, either by revisiting the literature, or using computational methods to optimize them.^35^

### Validation—External validity

We found that most of the models included a qualitative validation, meaning that simulation findings were mainly stated to agree with previously published ones. However, it is important to emphasize that qualitative validation cannot replace quantitative validation because it cannot demonstrate the predictability of a model. Quantitative methods can use real-life metabolic findings at the nanoscale taken from ^13^C or ^14^C labeling data^61,62^ or magnetic resonance spectroscopy^38^ from the literature as measures of key variables in the models such as glucose metabolic rate. Models that included the mesoscale can validate their glucose metabolic rate outputs using various available human FDG–PET datasets.

### Evaluation—Value added to the understanding of cerebral metabolism

The evaluation stage of the VV&E assesses whether value is truly added with the implementation of a neuro-metabolic model. Overall, we observed an emphasis on technical details of the computations and qualitatively reporting whether their predictions aligned with previously published models or wet-lab findings. Moreover, it was never clear whether the aim of choosing a complex version of the mechanism was to demonstrate the applicability of a computational method in a metabolic setting, or that the authors believed this was a realistic enough representation of cerebral metabolism in the chosen scale(s).

Performing sensitivity analysis can help identify and eliminate entities and respective parts of the mechanism that exert the weakest changes on the model’s outputs and make the model more efficient and reliable. At that point, the fluxomic approach may become more viable since it can better account for the highly dynamic and volatile nature of the brain given the abundance and dynamic nature of cerebral proteins.

Considering that the brain is tremendously robust in staying functional through decades of pathological insults from neurodegenerative diseases, it is reasonable to assume that it has an underlying resilience to remain homeostatically steady for most of its lifespan.^32^ By implementing more pro-trophic assumptions, a model could be less restricted to match static stoichiometric constraints and instead include metabolic phenomena that are more representative results of the brain’s trophic needs. It could redirect the focus on entities that keep the net metabolic efficiency at a physiologically healthy level. In addition to pro-trophic phenomena, the model can be modulated with atrophic metabolic entities known to be associated with aged or neurodegenerative disease individuals, which can trigger the neuro-inflammatory system.

### A multi-scale, multidisciplinary proposal

The prevalent tendency of these models to include nano and micro levels seemed to imply that a homogeneous brain with identical molecular activity across all cells, cell types, brain regions and networks is a good enough place to start. However, if we choose to focus a model on metabolic pathways (i.e. the nanoscale), we are bound to acknowledge the complexity and confusions of proteomic activities that come with it, when in fact, modeling cerebral metabolism does not have to be a bottom-up approach.^32^ Given that advanced mass profiling methods of protein have not yet helped grasp the nature of their functions,^63^ it raises the need to begin looking at knowledge beyond the nanoscale.

The benefit of looking at mesoscale findings such as brain imaging is the much broader availability of human brain images, which bypasses the translational gap from non-human to human physiologies that exist at the nanoscale. For example, live human brain FDG–PET data could be used as parametrization inputs as proxy measurements for the rate of brain glucose uptake.^64^

Higher scale equations can be classified accordingly based on their respective temporal scales which also helps prevent solver stiffness, known as a computational burden, that arises when simulations include very fast (e.g. micro or nanoseconds) and relatively much slower (e.g. years) phenomena in the same model.^64^

In summary, taking into account higher scales allows us to extend the model with higher-order phenomena that will impact one’s metabolic trajectory in one way or the other, beyond minutes or hours.^65^ Importantly, it can also help generate a model that can be applicable in real-life demands such as diagnosing neuro-metabolic diseases and searching for related therapeutics.^32^

### Limitations

We have limited our search to the PubMed database; therefore, we may have missed other computational models of glucose cerebral metabolism. We used exact search words, rather than using Medical Subject Headings (MeSH) terms or synonyms, which inevitably implies that some worthwhile publications were missed.

## Conclusion

We performed a high-level qualitative analysis of 15 articles reporting on computational models of brain metabolism. We emphasized what aspects of the reviewed models could have been improved to optimize their reproducibility and verifiability. We believe these considerations are important for the models to be further developed and become applicable for neuroscientists. Implementing the VV&E paradigm in the assessment of each model revealed that it is essential for authors to perform a mindful assessment of biological relevance, as it will help identify key areas of the design process and prioritize rational and more sensible assumptions over existing habitual misconceptions in mathematical or biological norms.

## Supplemental Material

sj-docx-1-jcb-10.1177_0271678X261465840 – Supplemental material for A scoping review of computational models on human glucose cerebral metabolism Supplemental material, sj-docx-1-jcb-10.1177_0271678X261465840 for A scoping review of computational models on human glucose cerebral metabolism by Parissa Fereydouni-Forouzandeh, Andréanne Michaud, Nicolas Doyon and Simon Duchesne in Journal of Cerebral Blood Flow & Metabolism
