# Supplementary material for: A scoping review of computational models on human glucose cerebral metabolism
Source: J Cereb Blood Flow Metab. 2026 Jun 24:0271678X261465840. Online ahead of print. doi: 10.1177/0271678X261465840 (PMC13380646; doi:10.1177/0271678X261465840)
Supplement: sj-docx-1-jcb-10.1177_0271678X261465840 – Supplemental material for A scoping review of computational models on human glucose cerebral metabolism [file sj-docx-1-jcb-10.1177_0271678X261465840.docx]

**SUPPLEMENTARY MATERIAL**

**Table S1.** Lexicon of key terms and contextualized meanings

| Terms | Meaning |
| --- | --- |
| Entity | Represents a dependent variable of interest defined by an equation (e.g., glucose or rate of change of glucose). |
| Output | The value of a dependent variable when running an equation in the model. |
| Scale | Spatial and temporal scope of model entities. |
| Nanoscale | Entities sized in nanometer range, measurable functions in milliseconds to minutes, and housed in a single cell compartment like a neuron or astrocyte (e.g., micro-molecules like glucose, lactate, ATP; as well as macro-molecules like hexokinase, glucose transporters). |
| Microscale | Entities sized in micrometer or voxel range, changes measurable in minutes to years (e.g., neuronal or astrocytic concentration). |
| Mesoscale | Entities at the organ level, observable with medical imaging, changes measurable in years (e.g., whole brain, brain segments, liver, adipose tissue). |
| Assumptions | Written or implied set of conditions, supposed to be true, which reflects the decision-making of model designers regarding concepts, methodological paths, or parameter values for which there is a lack of collective knowledge or unanimity. Careful and logical reasoning in the selection of assumptions, accompanied with all due explanations would thus enhance mathematical and biological plausibility, better able to produce a sensible neurobiological model which is understandable by various relevant disciplines. It can also minimize future unnecessary ambiguities or confusions, leading to more interpretable outcome predictions. |
| Core assumptions | Set of high-level assumptions carrying a pivotal role in the plausibility and overall predictability of the model, especially concerning conceptual and methodological designs. Therefore, the more the system of interest presents with uncertainties and confusions, such as metabolism in the complex and dynamic brain environment, the more it requires its core assumptions, as well as its potential implications on model outputs to be clear, reasonable and plausible. |
| Homogenous environment | An example of core assumption that metabolic processes occur uniformly throughout a given environment. |
| Steady-state | Also known as an equilibrium state, an example of a core assumption that the brain's biochemical reactions are in a state for which no change will occur unless the system receives an external perturbation. |
| Flux-balance analysis | In conjunction with the steady-state, the flux-balance analysis is an *in silico* computational method aiming to measure the rate at which these reactions return to steady-state when a disturbance is induced into the system. |
| Compartment | Semantic categorization of entities into homogenized environments within the system of cerebral metabolism. Compartments also imply that they house entities from a smaller scale (e.g., the glucose entity behaves homogeneously throughout the neuron compartment. See Figure 1B for a list of compartments). |
| Parameter | A measurable quantity that helps define the behavior of the dependent variable. It is a constant or varying quantity corresponding to a biological or biophysical entity such as the rate of a metabolic reaction. |
| Parameter source | Refers to the literature source from which a parameter was taken or estimated. The parameter values can sometimes be found directly in literature or may be adapted by the authors if the experimental context of the source differed from the context of the model. More specifically, we verify if the value was taken from human or non-human samples. |
| Initial condition | Considered as an input to the model, it is the state of the model at the beginning of the period of interest. |
| Initial condition source | Refers to the literature source from which an initial condition was taken or estimated. We verify if the value was taken from human or non-human samples. |
| Ordinary differential equation | Function depicting the rate of change of a single independent variable. |
| Partial differential equation | Function depicting the rate of change of multiple independent variables describing quantities that continuously depend on spatial coordinates. Neurobiological models using this type of equations typically assume a diffusive pattern of change throughout the geometry of the brain. |
| Model diagram | Schematic that summarizes key entities of focus in a model, as well as the main biological and/or physiological relationships between them (e.g., with regulatory arrows for positive and negative feedback effects, or substrates yielding products in a biochemical pathway). |
| Diagram complexity | A general assessment of the complexity of the model accounting for the number of entities considered. Model diagrams with a higher number of entities are considered more complex, which reflect a more complex system of equations. |
| Biological relevance | The overall biological soundness and conceptual validity of a model, in reference to its core assumptions. It indicates that the general tendencies of the model outcomes follow what is expected to occur biologically, such as a rise in blood glucose after eating a meal. |
| Methodological relevance | The overall mathematical soundness of a model with respect to its computational methodology. |
| Internal validity | Whether it is possible to reproduce the model’s results based on the equations and methodology provided. |
| External validity | Whether the outcomes of the model have been shown to replicate or closely align with published real-life experimental data. |
| Qualitative validation | The qualitative reporting of how closely the model outputs match or do not match real-life data as a result of external validation. |
| Quantitative validation | Numerical comparison between the model outcome and experimental data by statistical analysis. It determines how closely model outputs match, or do not match real-life data during external validation. |
| Validation with literature | External validation of model outputs with real-life data taken from the literature. |
| Validation with in-house data | External validation of model outputs with real-life data taken from experimental measurements obtained by the authors (whether *in vivo*, *in vitro* or *in silico*). |
| Model extensibility | An assessment of how easy it is to modify the model. We consider a model to be extensible if it fits the VV&E (Figure 2) criteria. It should therefore have the following prerequisite characteristics: basic reproducibility information, biological and methodological relevance, internal and external validation, and capacity to increase knowledge towards improving our understanding of important biological questions, as well as a related disease. |
